# An evaluation index system for regional mobile SARS-CoV-2 virus nucleic acid testing capacity in China: a modified Delphi consensus study

**DOI:** 10.1186/s12913-022-08446-9

**Published:** 2022-08-24

**Authors:** Dong-sheng Di, Jian-li Zhang, Mu-hong Wei, Hao-long Zhou, Yuan Cui, Ru-yi Zhang, Ye-qing Tong, Jun-an Liu, Qi Wang

**Affiliations:** 1 MOE Key Lab of Environment and Health, Department of Epidemiology and Biostatistics, School of Public Health, Tongji Medical College, Huazhong University of Science and Technology, Wuhan, 430030 China; 2 Hubei Provincial Center for Disease Control and Prevention, Wuhan, 430079 Hubei China; 3 Department of Social Medicine and Health Management, School of Public Health, Tongji Medical College, Huazhong University of Science and Technology, Wuhan, 430030 China

**Keywords:** Delphi method, Analytic hierarchy process, Nucleic acid testing, Testing capacity evaluation

## Abstract

**Background:**

Large-scale detection has great potential to bring benefits for containing the COVID-19 epidemic and supporting the government in reopening economic activities. Evaluating the true regional mobile severe acute respiratory syndrome coronavirus 2 (SARS-CoV-2) virus nucleic acid testing capacity is essential to improve the overall fighting performance against this epidemic and maintain economic development. However, such a tool is not available in this issue. We aimed to establish an evaluation index system for assessing the regional mobile SARS-CoV-2 virus nucleic acid testing capacity and provide suggestions for improving the capacity level.

**Methods:**

The initial version of the evaluation index system was identified based on massive literature and expert interviews. The Delphi method questionnaire was designed and 30 experts were consulted in two rounds of questionnaire to select and revise indexes at all three levels. The Analytic Hierarchy Process method was used to calculate the weight of indexes at all three levels.

**Results:**

The evaluation index system for assessing the regional mobile SARS-CoV-2 virus nucleic acid testing capacity, including 5 first-level indexes, 17 second-level indexes, and 90 third-level indexes. The response rates of questionnaires delivered in the two rounds of consultation were 100 and 96.7%. Furthermore, the authority coefficient of 30 experts was 0.71. Kendall’s coordination coefficient differences were statistically significant (*P* < 0.001). The weighted values of capacity indexes were established at all levels according to the consistency test, demonstrating that ‘Personnel team construction’ (0.2046) came first amongst the five first-level indexes, followed by ‘Laboratory performance building and maintenance’ (0.2023), ‘Emergency response guarantee’ (0.1989), ‘Information management system for nucleic acid testing resources’ (0.1982) and ‘Regional mobile nucleic acid testing emergency response system construction’ (0.1959).

**Conclusion:**

The evaluation system for assessing the regional mobile SARS-CoV-2 virus nucleic acid testing capacity puts forward a specific, objective, and quantifiable evaluation criterion. The evaluation system can act as a tool for diversified subjects to find the weak links and loopholes. It also provides a measurable basis for authorities to improve nucleic acid testing capabilities.

## Background

Coronavirus disease 2019 (COVID-19) [1] is an infectious disease caused by severe acute respiratory syndrome coronavirus 2 (SARS-CoV-2) [2]. Since the disease was first reported, the virus has rapidly spread globally [3], leading to the coronavirus pandemic [4] and a severe global recession [5]. The global spread of the virus has been drastic; in particular, more than 243 million cases have been confirmed in more than 200 countries, including 4.9 million deaths, as of 26 October 2021 [6]. The infection can be spread by asymptomatic, presymptomatic, and symptomatic infectors [7]. The Chinese government implemented unprecedented nonpharmaceutical public health measures in the early stages of the COVID-19 outbreak to control the local spread of COVID-19 and stabilise the epidemic. The SARS-Cov-2 virus has undergone several mutations, whilst the epidemic has been expanding worldwide, resulting in virulence alterations that impact illness severity around the world [8]. The introduction of vaccines against COVID-19 globally and in China has undoubtedly improved epidemiological situations. Nevertheless, extensive data suggests that the immune protection conferred by vaccines declines over time, allowing for the emergence of new diseases [9, 10].

Given the above background, the possibility that an epidemic will occur in vaccinated populations remains high because of the emergence of new SARS-CoV-2 variants and postvaccination infection [11]. Imported SARS-CoV-2 infectors and/or contaminated commodities from abroad would bring the potential risk of the local COVID-19 epidemic in China. Analysing respiratory discharges using real-time quantitative polymerase chain reaction is the most reliable method for virus detection [12]. The goal of ‘early discovery, early reporting, early isolation, and early treatment’ necessitates rapid and precise testing to respond effectively to the epidemic. Early data from the China epidemic supported the idea that effective and prompt testing would reduce the time between the start of symptoms and diagnosis, thereby lowering the number of severe and critical cases [13]. Besides, data from Brazil also emphasized that identifying interval time could favour efficiently carrying out prevention actions to contain the COVID-19 pandemic [14]. In summary, the case for mass, community-wide polymerase chain reaction (PCR) testing for COVID-19 of the right individuals at the right time remains strong.

Since the containment strategy against COVID-19 has been adopted in China, the measure of large-scale PCR-based testing of SARS-CoV-2 in throat swab samples is critical for sustaining containment in mainland China [15], particularly for controlling numerous local outbreaks caused by imported viruses. For example, between 11 June and 14 July, 2020, 11.9 million persons were tested at Beijing’s Xinfadi market during the outbreak [16], contributing to an optimal balance between epidemic containment and economic protection in Beijing [17]. In another two cases of COVID-19 epidemic control, 4.5 million and 10.9 million people received nucleic acid testing for SARS-CoV-2 virus within 5 days in Dalian [18] and Qingdao [19], respectively. Guangzhou performed a large-scale nucleic acid testing amongst 18.7 million people within 3 days to control the spread of COVID-19 [20]. Between 8 and 21 August 2020, a comprehensive community testing approach was performed in Vietnam, together with an innovative sample pooling mechanism, contributing to the ultimate success of COVID-19 control in Da Nang City [21]. New Zealand’s efficient mass testing contributed to the government’s success in curbing the spread of the coronavirus [22]. The significant number of asymptomatic individuals and evidence of substantial presymptomatic transmission highlighted the efficacy of mass testing in controlling the illness [22]. Timely and effective large-scale detection has great potential to bring benefits for containing the epidemic and supporting the government in reopening economic activities [23, 24].

Comprehensive, active and innovative PCR testing strategies need heightened requirements for nucleic acid detection capability to respond effectively to the epidemic that may be caused by new SARS-Cov-2 virus variants. On 2 September 2020, the State Council’s interagency task force released the Plan on Advancing Coronavirus Nucleic Acid Testing Capacity Building (the Plan) [25]. According to the Plan, regional mobile nucleic acid testing capacity is needed to ensure a highly responsive mobilisation mechanism; thus, all individuals residing in corresponding locations can receive nucleic acid testing within a short period in cases when local COVID-19 patients (either symptomatic or asymptomatic ones) are discovered in routine screening measures under the government’s regular epidemic prevention strategies. Finding the weak links and loopholes is critical in building nucleic acid testing capacity against COVID-19. However, no comprehensive and practical evaluation tools are available for relevant departments to find and strengthen the weak links of testing capacity in different regions.

Previous studies reported several challenges encountered in a massive nucleic acid testing [26–28]. Firstly, the time-consuming detection period and expensive testing reagents are the main limitations of a massive testing. In particular, the high cost of the detection reagent needed in a massive testing causes stress to a region’s economic budget [26]. The equipment for nucleic acid testing is in short because of the concurrent increase in global requirements. Secondly, a vast workforce is needed to maintain the program. The effectiveness and scope of this program to contain the outbreak also depend on a comprehensive national strategy [27]. In addition, the close contact between medical staff and COVID-19 infectors in throat swab collection increases the risk of virus infection for the staff [28]. Compared with first-tier cities, such as Beijing and Guangzhou, third- and fourth-tier cities have a relatively poor response and reaction ability in handling public health emergencies. Cross-infection of SARS-CoV-2 occurred in the several rounds of massive testing against the COVID-19 outbreak in Yangzhou, China, because of the lack of experience, poor planning and management [29]. Given the evidence mentioned above, the primary goal of this study was to provide a scientific and comprehensive evaluation index system of regional mobile nucleic acid testing capacity. This index system will help optimise relevant issues in the whole process of massive testing, ensure the safety of working staff and subjects, and avoid cross-infection. Additionally, our study provides evidence for large-scale nucleic acid detection management in other countries.

## Methods

### Overview of the research process

**Fig. 1:**
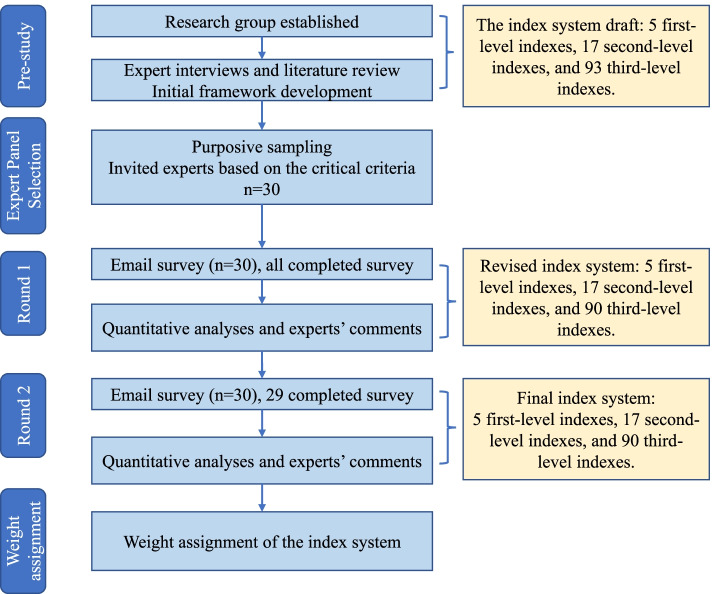
Flow diagram of modified Delphi study process

The detailed research process was shown in Fig. 1. In this work, we employed a modified Delphi technique [30, 31] to solicit input from experts on what should be used and prioritised as assessment indices of testing capacity in the execution of large-scale COVID-19 infection screening at the community level. We formed a research group that included an epidemiologist, a health management expert, three doctorate students, and three master’s degree applicants. The research team was also in charge of creating an initial draft of the evaluation index system for the regional mobile testing capacity of SARS-CoV-2 virus nucleic acid, recruiting experts and supplying associated materials, reviewing expert suggestions and making necessary changes. We conducted the whole research in three steps: (1) Producing an initial draft for subsequent consultations: our research team advocated and drafted the initial version of the evaluation index system. We built a framework referring to related standards obtained via expert interviews and literature review; (2) Expert consultations using the modified Delphi method: a Delphi questionnaire was designed according to the above framework. The two-round Delphi survey was conducted via email between 18 March and 1 June, 2021; and (3) calculation of the weight of each index using the Analytic Hierarchy Process (AHP): the weight was calculated to understand the importance of each index in the regional mobile nucleic acid testing capacity evaluation of the whole system.

### Expert panel composition

We selected the experts by purposive sampling of those engaged in occupations/scientific fields related to infectious disease (e.g. COVID-19) prevention and control policies, strategies and measures. According to the nucleic acid testing needs, experts from different cities with strict epidemic control requirements were selected to ensure that they represent a wide array of approaches, practices, and backgrounds. No agreement existed on the number of panelists needed for a Delphi [32]. The typical Delphi panel size was 15–60 [31]. Evidence suggested that a panel size of 23 participants was necessary to stabilise the response characteristics in Delphi surveys [33]. Thus, we invited 30 experts based on the inclusion criteria and our resources to allow attrition. This number also guarantees that the study could continue smoothly and release financial pressure and labour cost. We recruited 30 experts based on the following criteria: (1) willingness to participate in this study, (2) a bachelor’s degree or higher, (3) work experience in epidemic prevention and control as a senior professional title or intermediate and above professional title and (4) engagement in health management, infectious/chronic disease prevention, hygienic detection, or health emergency for more than 5 years.

### Delphi questionnaire preparation

A semi-open questionnaire was prepared for expert consultation. It consists of three parts: (1) the general information about the panelists, (2) the experts’ self-evaluation table and (3) the main text of the evaluation index system. The first part was to collect information about the time length of working experience in infectious diseases prevention and control, educational background, and professional title. The second phase involved gathering information on the experts’ decision-making processes and knowledge of the research topic. The third step involved gathering information on the indexes’ relevance, operability, and sensitivity and the retention and deletion of relevant items and expert comments on the items. The ‘Importance’ dimension indicates the role of this index in reflecting regional mobile nucleic acid testing capacity. The ‘Operability’ dimension refers to the content covered by the index that can be implemented smoothly under actual conditions. Moreover, the ‘Sensitivity’ dimension refers to the content covered by the index that can distinguish the mobile nucleic acid testing capacity between regions.

### Evaluation index system draft construction

This study produced a complete and practicable evaluation index system for assessing regional mobile nucleic acid testing capacity to create a realistic and operational benchmark for future use at provincial, municipal and district-level government offices. We constructed the evaluation index system draft, considering three main aspects. Firstly, the experts from areas where large-scale nucleic acid testing was previously performed were consulted to assess the feasibility and usefulness of the established evaluation index method. Secondly, the Plan [25], the Protocol for Prevention and Control of COVID-19 (the Protocol) [34] and the Guidelines for Organization and Practice of Novel Coronavirus Nucleic Acid Mass Testing (the Guidelines) were referred to as the basis to ensure the scientific and authoritative integrity of the evaluation index system. Thirdly, the complete guiding opinions offered by experts and researchers in the published literature were employed as a reference base to maintain the scientific integrity of the evaluation index.

### Two-round Delphi consultation

In the two-round Delphi consultation, the panelists who met the inclusion criteria were consulted with the questionnaire delivered by email and were required to respond and send the filled questionnaire in 2 weeks. If needed, video calls were made to provide necessary explanations to the experts who had questions about the project or indexes.

Upon receiving the email, the panelists read a brief introduction to the study processes and definitions of the dimensions of ‘Importance’, ‘Operability’ and ‘Sensitivity’. First, they were required to assign scores to the three dimensions, adding up to 100. Then, they provided their contact information (name and email address), educational background and familiarity (e.g. working years) with the research content in the questionnaire. Then, the importance, operability and sensitivity judgments were assessed on a 10-point scale, with 1 indicating ‘absolutely disagree’ (not a relevant or appropriate index) and 10 indicating ‘certainly agree’ (relevant and appropriate index) to the assertions. The experts were also encouraged to provide any advice or comments on each index in the questionnaire, particularly if they disagreed with the drafted indexes or suggested additional indexes.

Indexes were considered to achieve consensus if the mean values of the importance, operability and sensitivity scores were all equal to or more than 7 with a coefficient of variation (CV) < 0.25. The indexes were removed directly in the case of < 7 mean scores of either two of the ‘importance’, ‘operability’, and ‘sensitivity’ dimensions or a CV of ≥0.25. We also deleted the indexes that were suggested to be moved by four or more experts. In other situations of different opinions, the indexes were further discussed. Additionally, the research group further discussed expert opinions and then increased, merged, or modified some indexes. Finally, the group summarised and illustrated all modifications and sent the revised evaluation index system for the next round of expert consultation. After the consultation, the indexes achieving consensus were included in the final script of the evaluation index system (with minor amendments for sense only).

### Reliability of the Delphi method

The positive coefficient was employed to show the enthusiasm and collaboration of specialists in the research regarding the response rate of the questionnaire. The questionnaire response rate was computed as the ratio of the number of completed questionnaires returned to the total number of questionnaires sent out. A response rate of 70% or above implied a high level of positivity amongst specialists [35].

The expert authority coefficient (Cr) was used to assess the validity of consultation results. It was calculated as the average of the sum of the scores indicating the expert’s familiarity with the consulting field (Cs) and the category of the basis for the expert’s consulting answers (Ca). The experts’ familiarity was evaluated on a five-point scale, with scores of 0.2, 0.4, 0.6, 0.8 and 1.0 from the lowest to the highest level of familiarity with the consulting field. The experts’ basis of the consultation was scored 0.8 in theoretical analysis, 0.6 in case of work experience, 0.4 in case of literature at home and abroad, and 0.2 in case of subjective judgment.

Kendall’s Concordance Coefficient (ω) was used to assess the level of coordination between expert viewpoints, with a range of 0 to 1 indicating low to high levels of coordination [31]. The Chi-square test was used to analyse the significance of the coordination coefficient. Statistically significant results indicated that expert opinions are well coordinated and the outcome is trustworthy.

### Weight assignment of evaluation index system

#### Building the model

The hierarchical structure was built according to the Delphi method’s requirements, including the target, criteria and scheme layers [36]. The target layer in this study was the testing capacity evaluation system of regional mobile nucleic acid. The criteria layers were the first-level indexes established in this study. The subcriteria layers were the second-level indexes established in this study. The program layers were the third-level indexes established in this study.

#### Constructing expert judgment matrix

Judgment matrix assignment is a crucial part of AHP [37]. It ensures the consistency of judgmental thinking. For each index, we calculated the mean scores for the ‘importance’, ‘operability’ and ‘sensitivity’ dimensions. Then we calculated an aggregated weighted score [30] reflecting the combination of ratings for importance, operability, and sensitivity, using the formula below.

$$\mathrm{aggregated}\ \mathrm{weighted}\ \mathrm{score}=0.39\times \mathrm{mean}\ \mathrm{importance}+0.36\times \mathrm{mean}\ \mathrm{operability}+0.25\times \mathrm{mean}\ \mathrm{sensitivity}$$

The weights of importance, operability, and sensitivity were calculated based on the experts’ decision-making and familiarity with the research content. The experts assigned probability scores to importance, operability, and sensitivity that added up to 100%. Then, the mean probability scores of importance, operability, and sensitivity were calculated as the corresponding weights. The indexes in each level were compared in pairs according to aggregated weighted scores. Based on the judgment of indexes in each level, the weights of these indexes were calculated using the APH method [37].

#### Weight assignment of the index system

The above hierarchical structure pattern and judgment matrix were fed into the Yaahp software for analysis [38]. The weights of the first-, second- and third-level indexes were calculated using the weights of the first-, second- and third-level indexes. The combined weight signified that the weight distribution of the superior indices should be considered when determining the weight. The combined weight was calculated as the continuous production of each level’s initial index weight.

### Statistical analyses

Each item was described using descriptive statistics such as mean, standard deviation, and CV; Expert opinion consensus and the calculation of the positive coefficient, authority coefficient, and coordination coefficients were used to test the Delphi method’s reliability and validity. The consistency rate (CR) was utilised to determine whether the matrix is consistent. All quantitative analyses used IBM SPSS Version 24. The hierarchical analysis was constructed based on the Yaahp software [38].

This study was classified as a service evaluation and did not require national research ethics committee approval (as advised by the Ethics Committee of Tongji Medical College of Huazhong University of Science and Technology). We certified that all applicable institutional and governmental regulations concerning the ethical use of human volunteers were followed during this research.

## Results

### Panel characteristics

In this study, we adopted two rounds of expert consultation, enrolling 30 experts in seven cities, including Beijing, Nanjing, Shenzhen, Ningbo, Zhengzhou, Yichang and Wuhan. Amongst the 30 experts, 20 (66.7%) had a doctorate, 8 (26.7%) had a master’s degree and 2 (6.7%) had a bachelor’s degree. Eighteen experts held a senior title, and 12 held a vice-senior title. The mean working years was 22.23 years (standard deviation: 10.54, median: 21.5, rang: 5–46) (Table 1).

**Table 1:** Characteristics of consulting experts using the modified Delphi method [n (%)]

Characteristics	Round 1 consultation	Round 2 consultation
N	30	29
Gender		
Male	24 (80.0)	23 (76.7)
Female	6 (20.0)	6 (23.3)
Educational background		
Bachelor degree	2 (6.7)	2 (6.9)
Master degree	8 (26.7)	8 (27.6)
Doctor degree	20 (66.7)	19 (65.5)
Professional title level		
Senior	18 (60.0)	17 (58.6)
Vice-senior	12 (40.0)	12 (41.4)
Working years		
5–9	3 (10.0)	3 (10.3)
10–19	9 (30.0)	8 (27.6)
20–29	10 (33.3)	10 (34.5)
30–39	6 (20.0)	6 (20.7)
≥ 40	2 (6.7)	2 (6.9)
Major		
Health inspection and quarantine	7 (24.1)	6 (20.7)
Health management	12 (40.0)	12 (41.4)
Prevention and treatment of chronic diseases	21 (70.0)	21 (72.4)
Prevention and control of infectious diseases	3 (10.0)	3 (10.3)
Health education	2 (6.7)	2 (6.9)
Medical security	1 (3.3)	1 (3.4)
Health emergency	1 (3.3)	1 (3.4)
Pharmacovigilance	1 (3.3)	1 (3.4)
Nutrition and food safety	1 (3.3)	1 (3.4)

### Reliability of the two-rounded Delphi consultations

The response rates of the two-round consultation were 100 and 96.7%. Moreover, all filled questionnaires collected were valid for subsequent analysis. The Cr was 0.71 with 0.81 Cs and 0.60 Ca. The experts involved in this study had high authority.

The overall coordination coefficients of importance, operability and sensitivity were 0.243, 0.158, and 0.129, respectively, in the first round (Table 2). In the second round, the overall coordination coefficients of importance, operability, and sensitivity were 0.249, 0.217 and 0.171. Kendall’s coordination coefficients were larger in the second round than those in the first round (all *P* < 0.001 by chi-square test).

**Table 2:** The result of expert opinions’ coordination degree

Consultation	Hierarchical level	Importance			Operability			Sensitivity		
		Kendall’s ω	*χ*^*2*^	*P*	Kendall’s ω	*χ*^*2*^	*P*	Kendall’s ω	*χ*^*2*^	*P*
Round 1	First-level	0.029	3.441	0.487	0.069	8.229	0.084	0.043	5.191	0.268
	Second-level	0.182	73.916	< 0.001	0.066	26.63	0.021	0.076	30.959	0.006
	Third-level	0.224	494.647	< 0.001	0.168	371.40	< 0.001	0.133	293.307	< 0.001
	Overall	0.243	653.795	< 0.001	0.158	424.652	< 0.001	0.129	346.789	< 0.001
Round 2	First-level	0.085	9.913	0.042	0.127	14.759	0.005	0.167	19.329	0.001
	Second-level	0.158	73.447	< 0.001	0.107	49.531	< 0.001	0.184	85.458	< 0.001
	Third-level	0.217	539.577	< 0.001	0.216	519.392	< 0.001	0.151	363.291	< 0.001
	Overall	0.249	775.325	< 0.001	0.217	651.699	< 0.001	0.171	513.160	< 0.001

### Initial draft of the evaluation index system

Based on massive literature and expert interviews, the research generated a list of potential factors (‘indexes’) to cover the actual common situation related to detection capability. These factors were refined in a series of meetings to develop the initial draft of the evaluation index system, including 5 first-level indexes, 17 second-level indexes, and 93 third-level indexes.

### Revisions to the draft after the first-round Delphi consultation

All of the panelists completed the expert consultation in the First Round. Nineteen (63.33%) experts suggested modifying the indexes and providing scores for each index at the three dimensions as ‘importance’, ‘operability’, and ‘sensitivity’. In particular, the modification suggestions included: (1) modifying the first-level index ‘Testing capacity building’ to ‘Laboratory performance building and maintenance’; (2) merging the second-level indexes of ‘Daily testing personnel’, ‘Reserve testing personnel’ and ‘Mobile response testing personnel’ into one, i.e. ‘Testing personnel’; (3) adding relevant second-level indexes about sampling personnel; (4) adding a third-level index, i.e. ‘Whether to establish an incentive and reward system for emergency task work’.

The means of importance, operability and sensitivity scores ranged from 6.83 to 9.50, 7.27 to 9.03 and 6.90 to 8.50, respectively (Table 3). The CVs of importance, operability and sensitivity scores ranged from 0.09 to 0.32, 0.11 to 0.28 and 0.16 to 0.26, respectively (Table 3). The minimum mean and maximum CVs were 6.83 and 0.32, respectively, in the third-level indexes’ importance scores (Table 3).

**Table 3:** The result of expert opinions’ concentration degree

Consultation	Hierarchical level	Importance		Operability		Sensitivity	
		Mean	CV	Mean	CV	Mean	CV
1st round	First-level	8.87–9.30	0.09–0.17	8.40–9.00	0.11–0.17	7.8–8.50	0.16–0.23
	Second-level	8.13–9.50	0.10–0.19	8.33–9.03	0.11–0.19	7.53–8.43	0.18–0.26
	Third-level	6.83–9.03	0.11–0.32	7.27–9.00	0.11–0.28	6.90–8.47	0.17–0.25
2nd round	First-level	8.93–9.33	0.07–0.10	8.53–8.97	0.07–0.12	8.00–8.64	0.07–0.09
	Second-level	8.71–9.41	0.06–0.11	8.40–9.00	0.07–0.11	7.66–8.79	0.10–0.16
	Third-level	8.11–9.33	0.05–0.15	7.93–9.07	0.07–0.15	7.55–8.76	0.09–0.18

*CV* Coefficient of variation

Considering the experts’ comments, the research team modified 12 indexes (1 first-level, 6 second-level and 5 third-level indexes), deleted 22 indexes (2 second-level and 20 third-level indexes), added 21 indexes (2 second-level and 19 third-level indexes) and merged 3 third-level indexes into 1. The revised version of the evaluation index system, consisting of 5 first-level, 17 second-level and 90 third-level indexes, was sent to the experts for a second-round consultation.

### Further revisions after the second-round Delphi consultation

One expert dropped out in the second-round consultation; thus, 29 experts completed the consultation. Six (20.69%) experts suggested minor amendments to the present version. All means of scores for the importance, operability and sensitivity were ≥ 7, and all CVs were < 0.25 (Table 3). Such results demonstrated a consensus of experts’ opinions on the scores of the indexes at the three dimensions.

Eight third-level indicators were with minor changes based on the results of the questionnaires and group talks. The evaluation system for assessing the regional mobile nucleic acid testing capacity was constructed with 5 first-level, 17 second-level, and 90 third-level indexes (Table 4).

**Table 4:** The weight coefficient of each index in the evaluation system

First-level indexes	Weight coefficient	Second-level indexes	Weight coefficient	Third-level indexes	Weight coefficient	Combination weights
1. Nucleic acid mobile detection emergency system construction	0.1959	1.1 Emergency system for nucleic acid testing	0.3266	1.1.1 Whether the jurisdiction has established nucleic acid testing emergency leading group	0.3316	0.0212
				1.1.2 Whether to establish mobile response team management and temporary allocation system	0.3303	0.0211
				1.1.3 Whether to establish a multi-department linkage system	0.3380	0.0216
		1.2 Emergency and technical plan for nucleic acid testing	0.3367	1.2.1 Whether to establish an emergency plan for large-scale nucleic acid testing	0.2565	0.0169
				1.2.2 Whether to organize the study of a large-scale nucleic acid testing plan	0.2256	0.0169
				1.2.3 Update cycle of the emergency plan for large-scale nucleic acid testing	0.2344	0.0155
				1.2.4 The awareness rate of staff to the work content involved in the plan	0.2535	0.0167
		1.3 Emergency exercise	0.3367	1.3.1 Whether to make emergency drill plan	0.33	0.0218
				1.3.2 Number of emergency drills carried out	0.33	0.0218
				1.3.3 Whether the effectiveness of emergency drills is evaluated	0.34	0.0224
2. Personnel team construction	0.2046	2.1 Sampling personnel	0.3337	2.1.1 Number of sampling personnel	0.3517	0.024
				2.1.2 Average working years of sampling personnel	0.3127	0.0229
				2.1.3 Average number of professional training sessions for sampling personnel	0.3356	0.0214
		2.2 Testing personnel	0.3381	2.2.1 Number of testing personnel	0.2619	0.0181
				2.2.2 Average working years of testing personnel	0.2391	0.0165
				2.2.3 Average number of professional training sessions for testing personnel	0.2475	0.0171
				2.2.4 Number of qualified qPCR staff	0.2515	0.0174
		2.3 Technically training	0.3282	2.3.1 Whether to develop a training plan for nucleic acid testing	0.1968	0.0132
				2.3.2 Number of nucleic acid testing training	0.1939	0.0130
				2.3.3 Number of offline training	0.2013	0.0135
				2.3.4 Whether the training content is assessed	0.2034	0.0137
				2.3.5 Pass rate of training assessment	0.2046	0.0137
3. Emergency response guarantee	0.1989	3.1 Financial guarantee	0.3423	3.1.1 Financial burden rate of dedicated funds	0.1669	0.0114
				3.1.2 Reserve funds	0.1679	0.0114
				3.1.3 Capital availability rate within one week	0.1692	0.0115
				3.1.4 Timeliness rate of funds within one week	0.1655	0.0113
				3.1.5 Budget execution rate	0.1612	0.0110
				3.1.6 Whether to establish an incentive and reward system for emergency task work	0.1692	0.0115
		3.2 Materials reserves	0.3371	3.2.1 Number of emergency vehicles	0.1398	0.0094
				3.2.2 Number of mobile laboratories	0.1390	0.0093
				3.2.3 Whether to establish a reserve catalogue of nucleic acid testing emergency materials	0.1470	0.0099
				3.2.4 Number of types of laboratory test materials stored	0.1433	0.0096
				3.2.5 Compliance rate of laboratory test materials stored	0.1467	0.0098
				3.2.6 Available time of sampling material in reserve	0.1415	0.0095
				3.2.7 Available time for testing material in reserve	0.1427	0.0096
		3.3 Material management	0.3207	3.3.1 Whether to establish a management record for emergency supplies for nucleic acid testing	0.3367	0.0215
				3.3.2 Integrity rate of management records of emergency supplies for nucleic acid testing	0.3340	0.0213
				3.3.3 Nucleic acid detection materials supplement and renewal cycle	0.3293	0.0210
4. Laboratory performance building and maintenauce	0.2023	4.1 Equipment configuration	0.1994	4.1.1 Compliance rate of testing equipment	0.1296	0.0052
				4.1.2 Normal operation rate of testing equipment	0.1292	0.0052
				4.1.3 Number of Nucleic Acid Extractors	0.1279	0.0052
				4.1.4 Number of PCR machines	0.1272	0.0051
				4.1.5 Number of biological safety cabinets	0.1250	0.0050
				4.1.6 Whether to establish instrument operation and maintenance records	0.1183	0.0048
				4.1.7 Integrity rate of operation record registration	0.1223	0.0049
				4.1.8 Integrity rate of maintenance record registration	0.1206	0.0049
		4.2 Specimen reception and management	0.1968	4.2.1 Integrity rate of specimen information registration	0.1298	0.0052
				4.2.2 Accuracy rate of specimen information registration	0.1308	0.0052
				4.2.3 Whether to set up a secimen bank of COVID-19 specimens	0.1273	0.0051
				4.2.4 Specimen bank capacity	0.1244	0.0050
				4.2. Number of refrigerators for specimen storage	0.1226	0.0049
				4.2.6 the sum of specimens received per day	0.1221	0.0049
				4.2.7 Storage time of each batch of specimens	0.1214	0.0048
				4.2.8 Backlog of experimental specimens	0.1214	0.0048
		4.3 Laboratory testing	0.1999	4.3.1 Sample pre-processing time	0.1675	0.0068
				4.3.2 Time spent on reagent preparation	0.1618	0.0065
				4.3.3 Time spent on Nucleic acid extraction	0.1635	0.0066
				4.3.4 Time spent on Nucleic acid amplification	0.1615	0.0065
				4.3.5 Average daily detection volume	0.1752	0.0071
				4.3.6 Average daily test volume in mobile laboratories	0.1705	0.0069
		4.4 Quality assurance and quality control	0.2025	4.4.1 Frequency of instrumentation calibration	0.1217	0.0050
				4.4.2 Inter-assay precision	0.1261	0.0052
				4.4.3 Intra-assay precision	0.1261	0.0052
				4.4.4 Limits of detection	0.1261	0.0052
				4.4.5 Contamination of high-concentration to low-concentration samples	0.1261	0.0052
				4.4.6 Contamination of low- concentration to high-concentration samples	0.1222	0.0050
				4.4.7 Frequency of internal and external quality controls	0.1256	0.0051
				4.4.8 Number of quality control failure events	0.1259	0.0052
		4.5 Laboratory biosafety management	0.2014	4.5.1 Whether to establish a laboratory environment disinfection record	0.1680	0.0068
				4.5.2 Integrity rate of laboratory disinfection records	0.1661	0.0068
				4.5.3 Laboratory environment disinfection frequency	0.1667	0.0068
				4.5.4 Biosafety self-inspection records	0.1667	0.0068
				4.5.5 Frequency of laboratory biosafety self-inspection	0.1641	0.0067
				4.5.6 Whether to establish a laboratory safety manual	0.1693	0.0069
5. Information management system for nucleic acid testing resources	0.1982	5.1 Technical personnel database	0.3331	5.1.1 Whether to establish an employee database	0.3421	0.0226
				5.1.2 Integrity rate of database personnel information registration	0.3266	0.0216
				5.1.3 Data update frequency of employee database	0.3313	0.0219
		5.2 Information management of nucleic acid testing	0.3325	5.2.1 Whether to establish a sampling reservation system	0.1347	0.0089
				5.2.2 Whether to establish a material resource information database	0.1369	0.0090
				5.2.3 Whether to equip the sampling point with scan code and take code device	0.1411	0.0093
				5.2.4 Whether the testing organization is equipped with a smart scan code data terminal	0.1400	0.0092
				5.2.5 Whether the sampling registration information and the agency testing information are interoperable	0.1472	0.0097
				5.2.6 Whether to establish an information management platform	0.1516	0.0100
				5.2.7 Whether the result of the test is connected with the health code	0.1485	0.0098
		5.3 Testing results presentation and feedback	0.3344	5.3.1 Whether to realize the electronic testing result report	0.1658	0.0110
				5.3.2 Average reporting time for fever and emergency patients	0.1712	0.0113
				5.3.3 Average reporting time in general outpatient clinics	0.1632	0.0108
				5.3.4 Average reporting time of hospitalization	0.1612	0.0107
				5.3.5 Average reporting time of the community that should be checked	0.1622	0.0107
				5.3.6 Average reporting time of positive results	0.1765	0.0117

### Capacity evaluation system weight distribution

In this study, 23 judgment matrices were constructed. These matrices had less than 0.1 CR values in the consistency test for all indexes, showing that the matrix’s degrees of inconsistency were scientifically acceptable. The weight coefficient for each of the evaluation indexes was calculated referring to the results of the consistency test, suggesting that ‘Personnel team construction’ with a weight coefficient of 0.2046 came first amongst the five first-level indexes, followed by ‘Laboratory performance building and maintenance’ (weight coefficient = 0.2023), ‘Emergency response guarantee’ (0.1989), ‘Information management system for nucleic acid testing resources’ (weight coefficient = 0.1982) and ‘Regional mobile nucleic acid testing emergency response system construction’ (weight coefficient = 0.1959) (Table 4).

## Discussion

The testing capability evaluation index system was built using a modified Delphi approach in this study, including 5 first-level indexes, i.e. ‘Personnel team construction’, ‘Laboratory performance building and maintenance’, ‘Emergency response guarantee’, ‘Information management system for nucleic acid testing resources’ and ‘Regional mobile nucleic acid testing emergency response system construction’, 17 second-level indexes and 90 third-level indexes. The AHP approach was used to calculate the weight coefficient for the three-level evaluation indexes. Moreover, the weight coefficient was reliable with consistency ratios of all less than 0.10. As policymakers worldwide seek to improve COVID-19 prevention, detection and response amid a flare-up of cases driven by the highly contagious Delta variant, the introduction of new nucleic acid testing programmers appears likely to continue. However, comprehensive and practical evaluation tools have not been available to monitor and evaluate nucleic acid testing capacity within and across jurisdictions. For the first time, the outcomes of this research provided robust references to benchmark mass testing capacity in China, which may then be refined for comparison with the outcomes from other countries developing and offering mass testing.

The evaluation index system for assessing regional mobile nucleic acid testing capacity was scientific, comprehensive and diversified with the following characteristics. Firstly, the theoretical basis was efficient and reliable. The initial draft of the evaluation index system was constructed based on the technical specifications and laws and regulations promulgated by the state and literature reviews, with reference to the Plan [25], the Protocol [34] and the Guidelines. In this study, relevant panelists were selected based on specific features (e.g. age, profession title and working year). These panellists were influential and active in COVID-19 epidemic prevention and control. Our team implemented revisions provided by specialists from many departments and perspectives, thereby ensuring that the system was suited for usage in various places with varying epidemic risk levels. Secondly, the authority coefficient (0.71) and questionnaire response rate (100 and 96.7%) were within the acceptable limits (more than or equal to 0.70) [35, 39]. Thirdly, this evaluation index system was systematic and comprehensive because we considered not only the whole process of nucleic acid detection but also the nucleic acid testing preparation, nucleic acid detection implementation and testing result presentation. Moreover, timely financial and material support and the construction of the emergency response system were considered. Fourthly, the diversified subject could use the evaluation index system to adjust and perfect the regional mobile novel coronavirus nucleic acid test activities. Each provincial or city-level government can conduct a self-assessment according to its situation. Besides, the government can evaluate the capacity of different areas according to the index. Diversified evaluation might assure fairness and impartiality in the overall nucleic acid testing capacity assessment.

The evaluation system followed the principles of integrity, emphasis, hierarchy, comparability and operability to achieve the purpose of ‘early detection, early reporting, early isolation, and early treatment’ of COVID-19 patients [40], and reduced the risk of large-scale spread of epidemics, by making arrangements in five aspects: regional mobile nucleic acid testing emergency response system construction, personnel team construction, emergency response guarantee, laboratory performance building and maintenance, information management system for nucleic acid testing resources. ‘Personnel team construction’ and ‘laboratory performance building and maintenance’ had the highest weights, indicating that they were important in strengthening the nucleic acid sampling and testing capacity in the personnel and laboratory techniques. Cross-contamination and other diagnostic mistakes, including those caused by an increase in specimens, a shortage of laboratory personnel, and a lack of quality control, may occur in a laboratory setting [41]. Stratigraphically speaking, our evaluation index system was efficient and reliable to some extent. COVID-19 testing on a large scale necessitates many people, many of whom may be untrained and working in a stressful environment. The regional mobile nucleic acid testing task necessitates a dynamic deployment of staff on duty in each part and a standby shift. For the emergency circumstance of COVID-19 mass testing, an emergency human resource allocation plan and a reserve echelon rotation mechanism must be developed, and proper rotation and collocation must be carried out [42]. As a result, the study group argued that long-term and standard human resource management procedures should be devised to ensure the smooth progression of the mass testing activity.

As for testing capacity, ‘Laboratory performance building and maintenance’ integrated the specimen reception, testing and biosafety management into a network that can respond effectively and efficiently to emergencies. Sufficient qualified personnel and efficient laboratory performance support provinces, cities and districts to provide increased sample analyses in epidemic outbreaks or other large-scale emergency events requiring surge capacity testing of samples and products. As reported, two air-inflated COVID-19 test laboratories, namely Huo-Yan Laboratory [43] and Falcon laboratory [44], were built in 1 day to meet the surge of testing demands in Guangzhou. Saudi Arabia, Brunei, and Kazakhstan are amongst the countries and territories that have started or built Huo-Yan labs to combat the COVID-19 virus [43]. New sampling and testing approaches have been described in recent studies [45–47], indicating that further training with drills and assessments is required to assure standard and uniform sample collection and testing by all employees.

The weight of ‘Emergency response guarantee’ in the first-level indexes was the third largest. The guarantee of emergency nucleic acid testing supplies was essential for medical personnel to implement emergency testing work and safety protection [48]. Therefore, the procurement and use of emergency supplies and other management work were directly related to the speed of response to the outbreak and the final effect of emergency testing work [49]. Perfecting the procurement, reserve and use management system of emergency nucleic acid testing supplies is the key to improving emergency management and handling capacity. The weight of ‘Information management system for nucleic acid testing resources’ in the first-level indexes followed ‘Emergency response guarantee’. Establishing an information management system for nucleic acid testing resources could comprehensively, accurately and dynamically grasp the information of nucleic acid testing institutions, sampling and testing personnel, thereby achieving efficient and accurate management of their deployment. The first-level index ‘Nucleic acid mobile detection emergency system construction’ had the lowest weight; however, this finding did not mean it was unimportant. Studies have found that mass gatherings organized in places with high population density can easily lead to widespread and cluster outbreaks of epidemic diseases, such as the cluster epidemic of Buddhist gatherings in Ningbo [50] and the outbreak epidemic in the health training centre of Jilin Province [51]. Once an outbreak occurs, the difficulty of epidemic prevention and control increases if all cases cannot be identified quickly. Therefore, when COVID-19 patients were reported, the nucleic acid mobile detection emergency system should be activated as soon as possible to prevent the epidemic from spreading.

For the second-level indexes, we adopted expert opinions to merge the ‘Daily testing personnel’, ‘Reserve testing personnel’, and ‘Mobile response testing personnel’ to ‘testing personnel’. We also added ‘Sampling personnel’ because proper specimen collection is the most important step in the laboratory diagnosis of infectious diseases. A specimen not collected correctly may lead to false or inconclusive test results. The novel coronavirus testing specimens shall be collected by qualified technicians who have received biosafety training (who have passed the training) and are equipped with the corresponding laboratory skills.

The time spent in a large-scale nucleic acid program is an important factor for taking further interventions to control infection spread. We considered this issue along two lines (both authorities and residents). For authorities, the main aim was to find the virus infectors and block transmission at the fastest speed. Thus, several periods (e.g. the detection time of nucleic acid) are critical for authorities to take decisive action, which is needed as soon as possible. For residents, the major concern is the time from throat swab collection to the release of the test result. Thus, we defined indexes for the ‘Testing results presentation and feedback’ (Table 4). The results’ reporting time for residents/patients was defined as the time from throat swab collection to the release of the test result. It can represent the total turnaround time (time from throat swab collection or arrival of the sample at the laboratory until the result is communicated to the patient/person or authorities). Consequently, the total turnaround time and phased time can be used to assess the regional nucleic acid testing capacity.

Some experts believed that the reward for emergency work should be quantified according to the workload of different areas, and the epidemic’s severity should be considered. The study group argued that the flexible index should be set with the goal of protecting the health of all employees in mind, and that it should be updated dynamically as the epidemic progresses.

In the setting of the global COVID-19 pandemic, our findings supported the application of evaluation index system in China. Moreover, generalising the evaluation index system to other countries was prudent. China is a special country in terms of health care because of its large population size and unbalanced health care development in different areas [52]. The Chinese government has been spending a lot of manpower and resources to overcome this public health issue. The large-scale nucleic acid testing strategies support China’s sustained containment of COVID-19, regardless of backward and developed regions in China. Consequently, according to China’s experience, this evaluation index system may be prudently extrapolated to many of other low- and middle-income countries, each one with its particularities.

Several strengths characterized our study. Firstly, this study was the first to construct the evaluation index system of regional mobile nucleic acid testing capacity. Thus, this study provided a reference for nucleic acid mobile detection capability evaluation. It also has guiding relevance for ensuring nucleic acid detection capability. Secondly, Delphi method allows the development of defensible, valid, and reasonable solutions based on expert opinion [31]. The Delphi method is regarded as a structured approach for evaluating and combining human judgment. Rowe et al stated that the Delphi method can be used when the researcher is convinced that the technique can generate more accurate assessments and judgments than that provided by individuals [53]. Our objective was to develop a comprehensive tool for evaluating the regional mobile SARS-CoV-2 virus nucleic acid testing capacity. Thus, we resorted to the Delphi technique. Considering the issues of seniority, interfering or inhibiting personality traits that easily occur in a face-to-face meeting, the Delphi method is reliable in obtaining an objective and unbiased point of view. The experts from regions with strict epidemic control requirements in different provinces and cities were selected to avoid bias in the judgment of approaches, practices, and backgrounds. Thirdly, we ensured anonymity between participants, avoiding the interplay effects. Fourthly, the items’ importance, operability and sensitivity were comprehensively considered in this study, indicating that the representativeness of weights was multidimensional and practically meaningful. However, this study also had limitations. Firstly, we realised that the decision-making process inevitably involved subjectivity and judgmental inputs in terms of panel selection, item selection and dispute resolution. Secondly, the initial outbreak of COVID-19 was in Wuhan; hence, a limitation may exist because approximately one-third of the experts were from Hubei. Furthermore, more than a third of the specialists invited were from research institutes. Their perspectives could not be more comprehensive than frontline healthcare staff.

## Conclusions

We established an evaluation index system for regional mobile nucleic acid testing capacity based on a scientifically designed Delphi process. Several important points obtained from this study. Firstly, the evaluation index system proposed specific, objective, and quantitative evaluation criteria that aid the government in containing the pandemic and resuming economic operations. In particular, the evaluation index system may help relevant departments to find and strengthen weak links of testing capacity in different regions. Secondly, the index weights informed the functional departments about the priority in managing a massive nucleic acid test. This evaluation index system may be generalised to other countries with prudence considering the shared, different, and complicated conditions in different countries. International consideration and feedback about the suitability of the evaluation index system are encouraged to develop an international consensus for virus nucleic acid testing against COVID-19.

## Data Availability

The datasets used and/or analyzed during the current study are available from the corresponding author on reasonable request. The data are not publicly available due to privacy or ethical restrictions.
